# Supplementary material for: Caerin 1.1/1.9 Enhances Antitumour Immunity by Activating the IFN-α Response Signalling Pathway of Tumour Macrophages
Source: Cancers (Basel). 2022 Nov 24;14(23):5785. doi: 10.3390/cancers14235785 (PMC9738106; doi:10.3390/cancers14235785)
Supplement: Supplementary file 1 [file cancers-14-05785-s001.zip › cancers-2005308-supplementary.pdf]

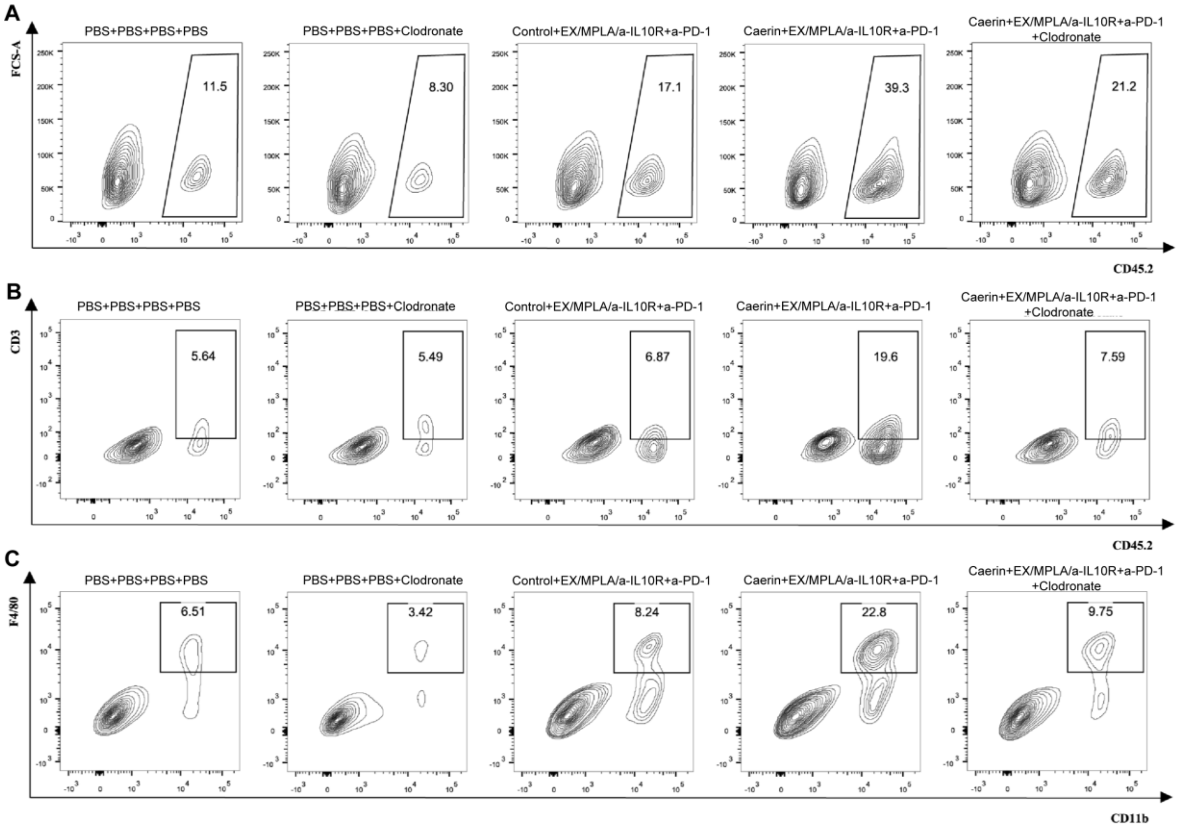

Figure S1. The tumours were dissected from the mice, digested into single-cell suspension and stained for analysis by flow cytometry. (A) The flow cytometric graphs of tumour infiltrating macrophages (CD45+CD11b+F4/80+), (B) The flow cytometric graphs of lymphocyte (CD45.2+) and (C) The flow cytometric graphs T cells (CD3+).

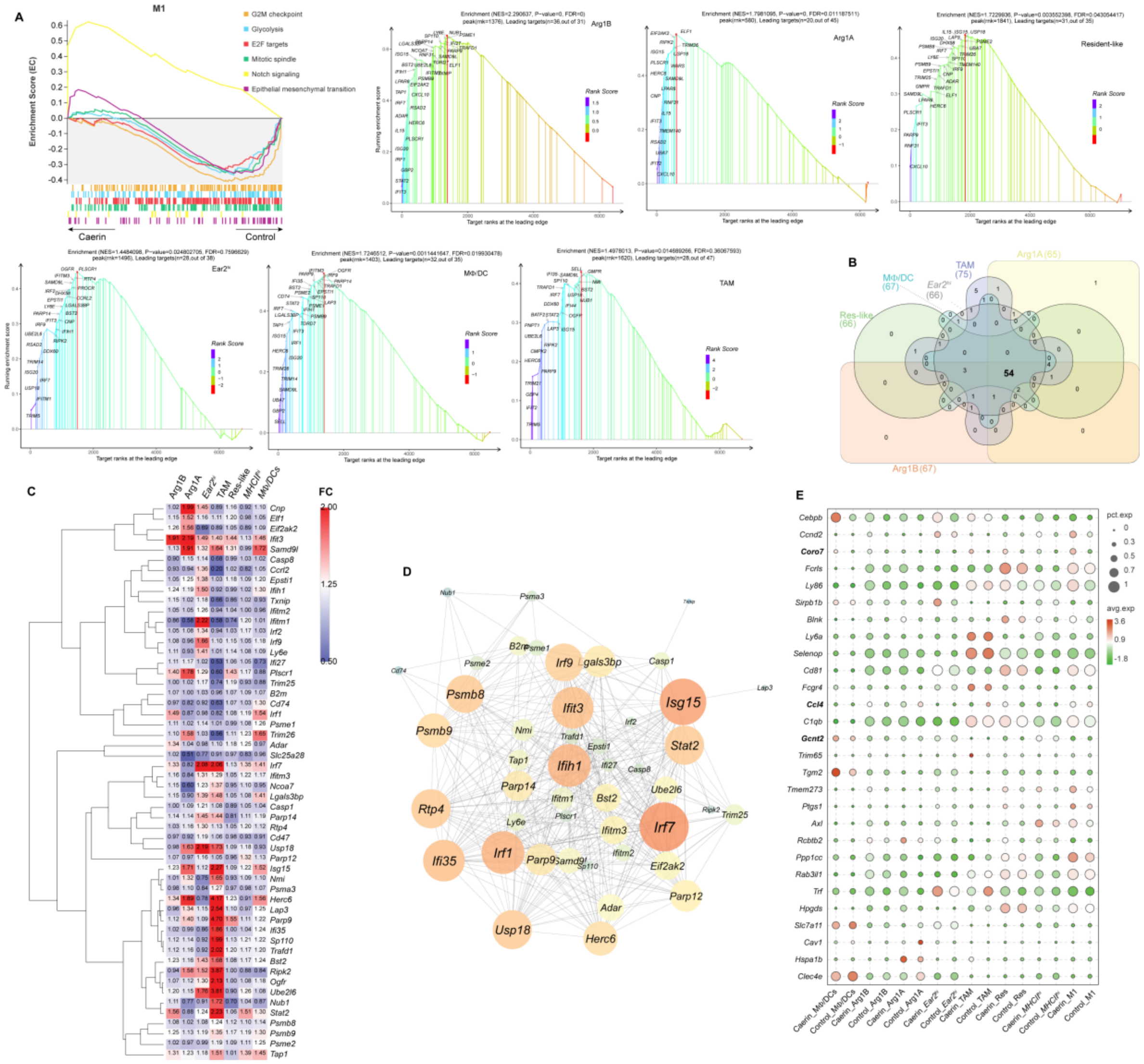

Figure S2. The comparative analysis of the enrichment of IFNα response signalling pathway in different phenotypes of macrophages with the topical application of the gel containing caerin 1.1/1.9. (A) The enrichment of the Hallmark pathways in the M1-like MΦs of the caerin and control groups, respectively, predicted by the GSEA analysis. The ranking of genes associated with the enrichment of IFNα response signalling pathway in the other MΦ populations, including Arg1B, Arg1A, resident-like, Ear2^{hi}, MΦ/DC and TAM. (B) The Venn diagram compares the DSGs identified on the IFNα response signalling pathway in different populations. (C) The expressions (in FC values) of the 54 DSGs identified in (B), in all MΦ populations of the caerin group with respect to the control. (D) The PPIs among the proteins derived from the 54 DSGs. (E) Comparison of the average expression of the top 30 DEGs (significantly in at least one MΦ population) in relative to their percentage of expression.

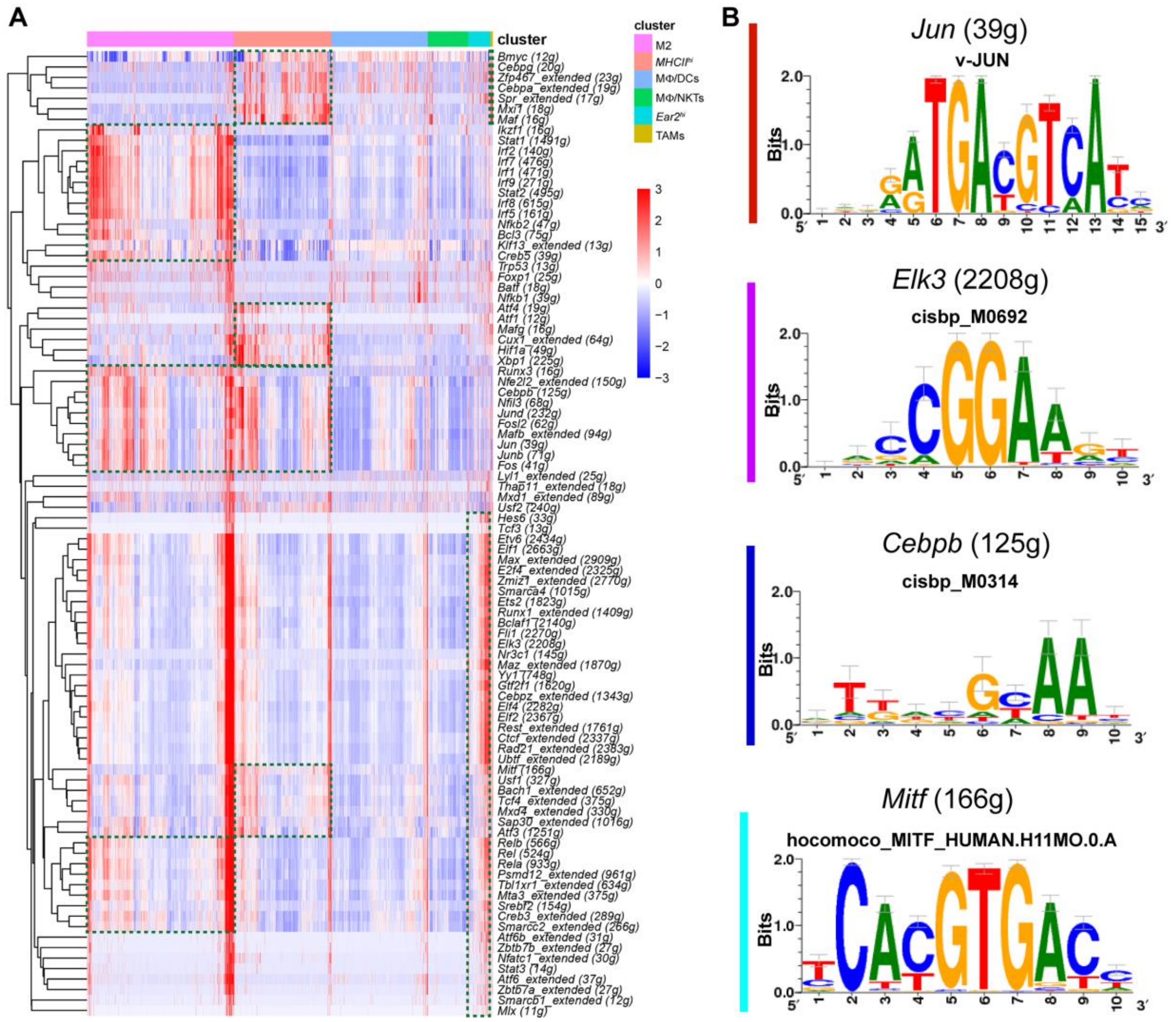

Figure S3. The regulation of MΦ populations in the triple therapy. (A) SCENIC analysis of the MΦs in the TME; differential regulators are circled. (B) The corresponding enriched DNA-binding motifs are shown (Logo); the regulators are colour matched with the TFs in Figure 4A.

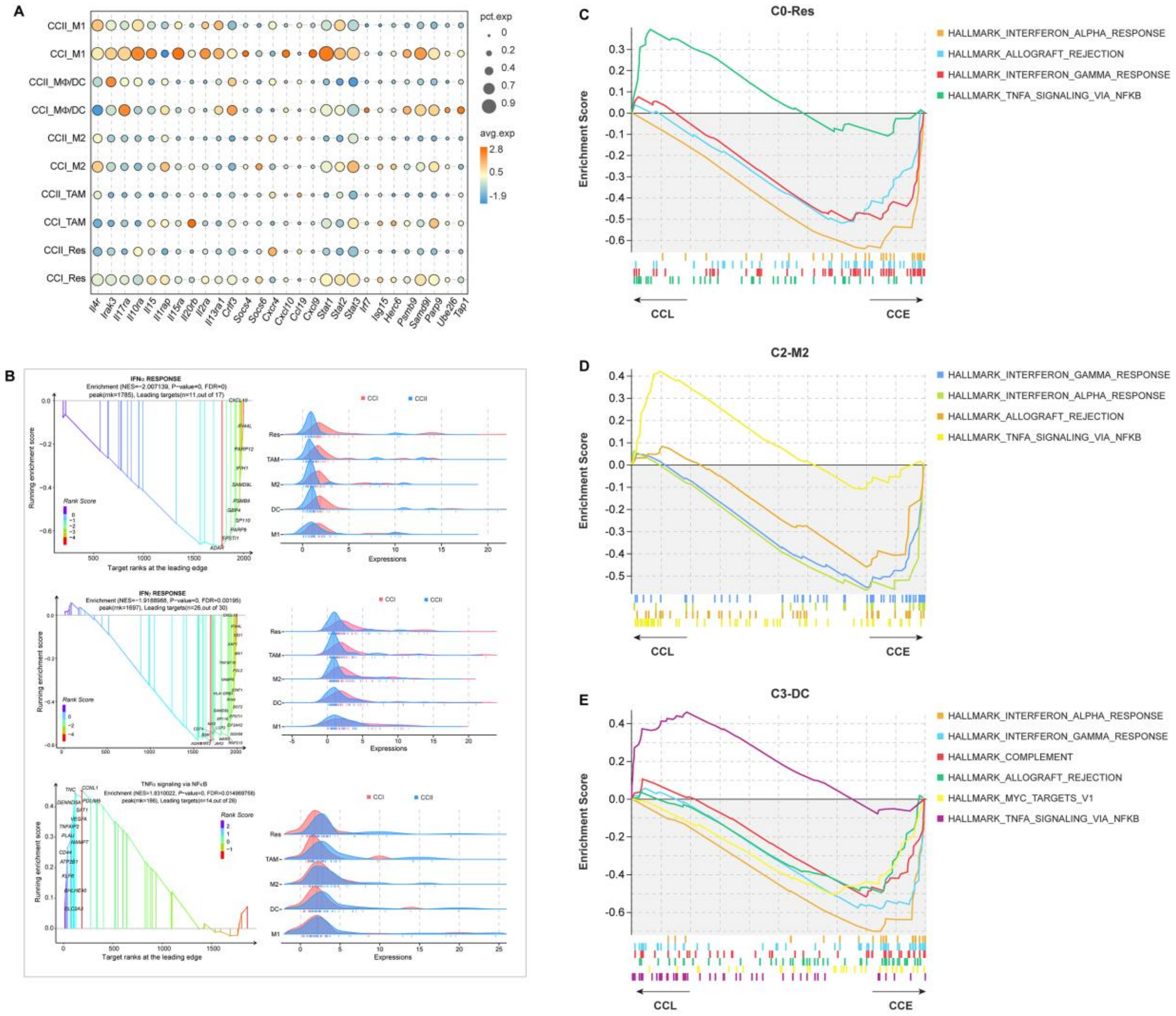

Figure S4. The comparison of interleukin and chemokine regulation and the pathways enriched in the MF populations of the CCI and CCII groups. (A) The DSGs of interleukin and chemokine regulations in different MF phenotypes, including M1-like, MΦ/DC, M2-like, TAM and resident-like MFs. (B) The activation of IFN-α and IFN-γ response pathways was present in all MΦ phenotypes of the CCI group, while activation of TNF-α was detected in all MF phenotypes of the CCII group, except M1-like MΦs. (C) The comparative analysis of the Hallmark pathways enriched in the resident-like (C), M2-like (D) and MΦ/DC (E) populations of CCI and CCII groups, respectively.
