# Supplementary figures and images for: Caerin 1.1/1.9 Enhances Antitumour Immunity by Activating the IFN-α Response Signalling Pathway of Tumour Macrophages
Source: Cancers (Basel). 2022 Nov 24;14(23):5785. doi: 10.3390/cancers14235785 (PMC9738106; doi:10.3390/cancers14235785)

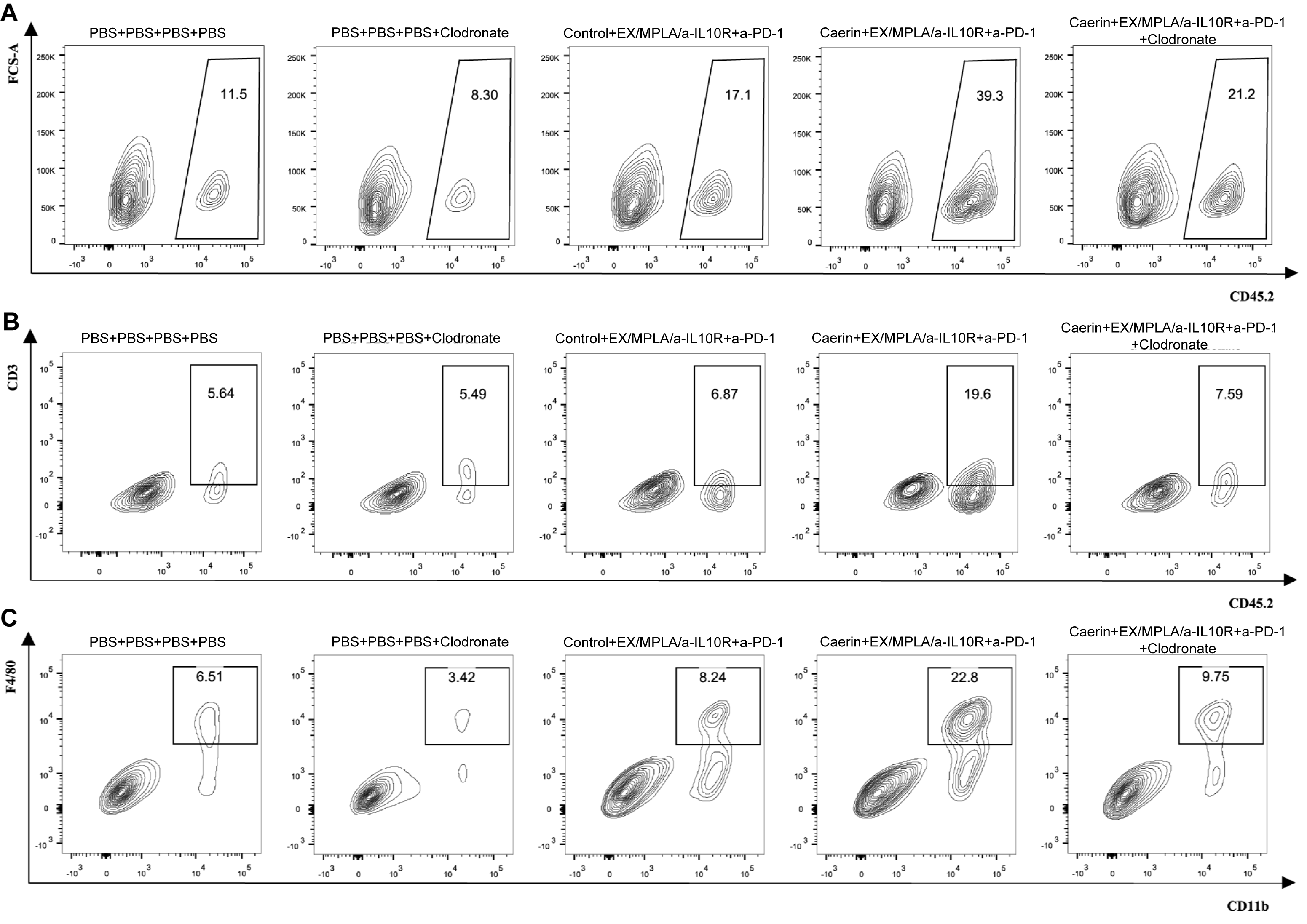

Supplement: Supplementary file 1 [file cancers-14-05785-s001.zip › Figure S1.tif]

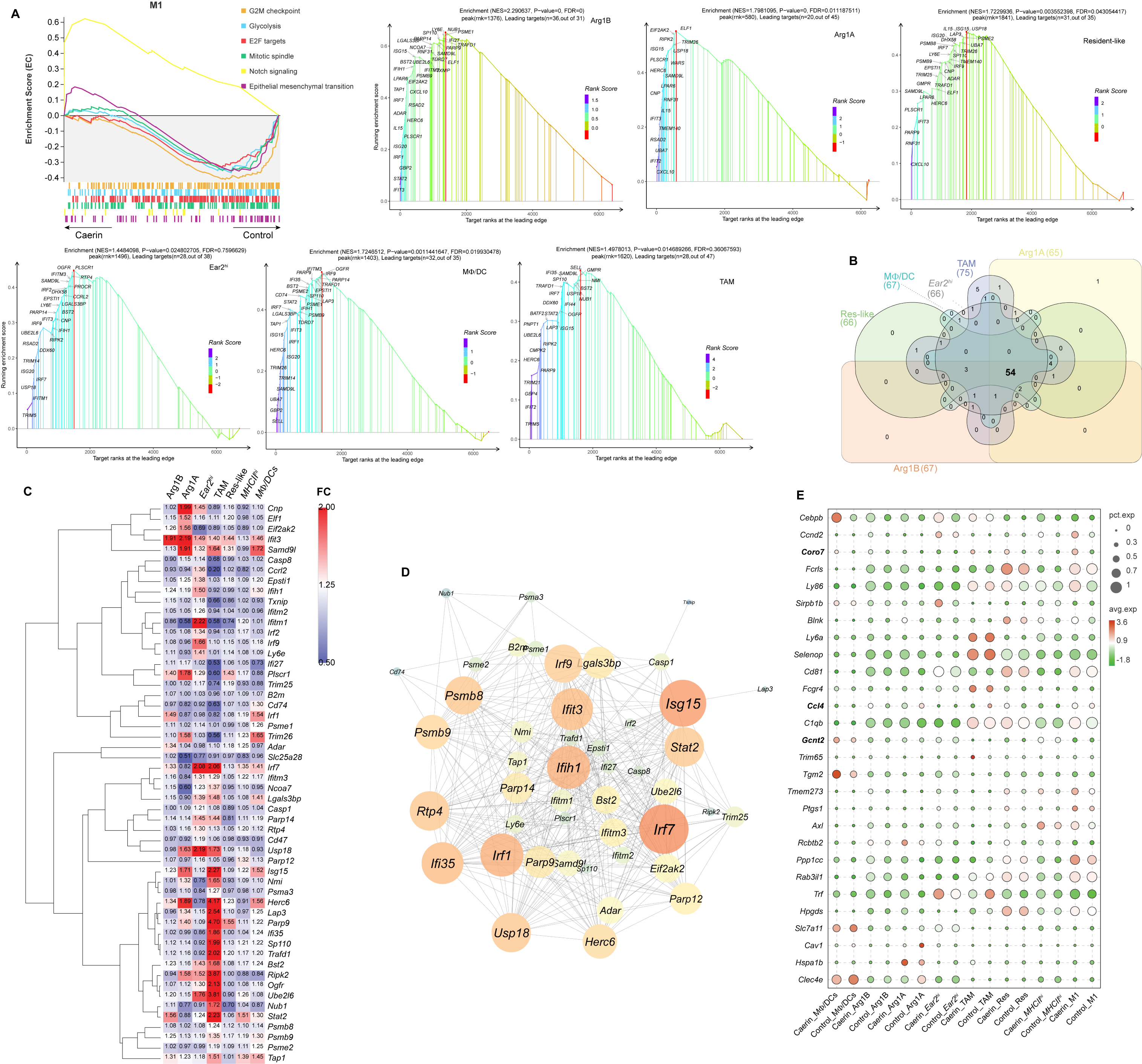

Supplement: Supplementary file 1 [file cancers-14-05785-s001.zip › Figure S2.tif]

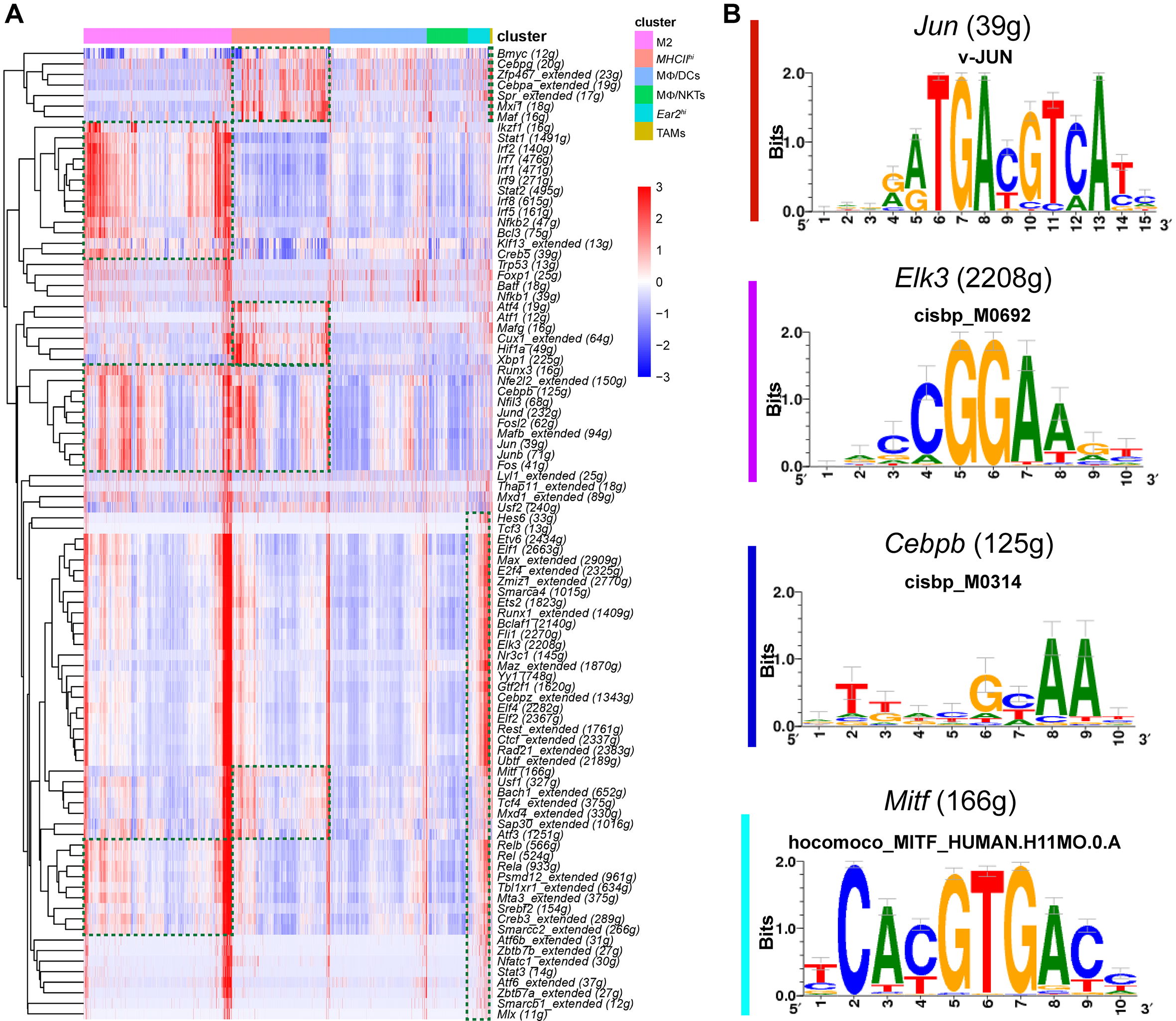

Supplement: Supplementary file 1 [file cancers-14-05785-s001.zip › Figure S3.tif]

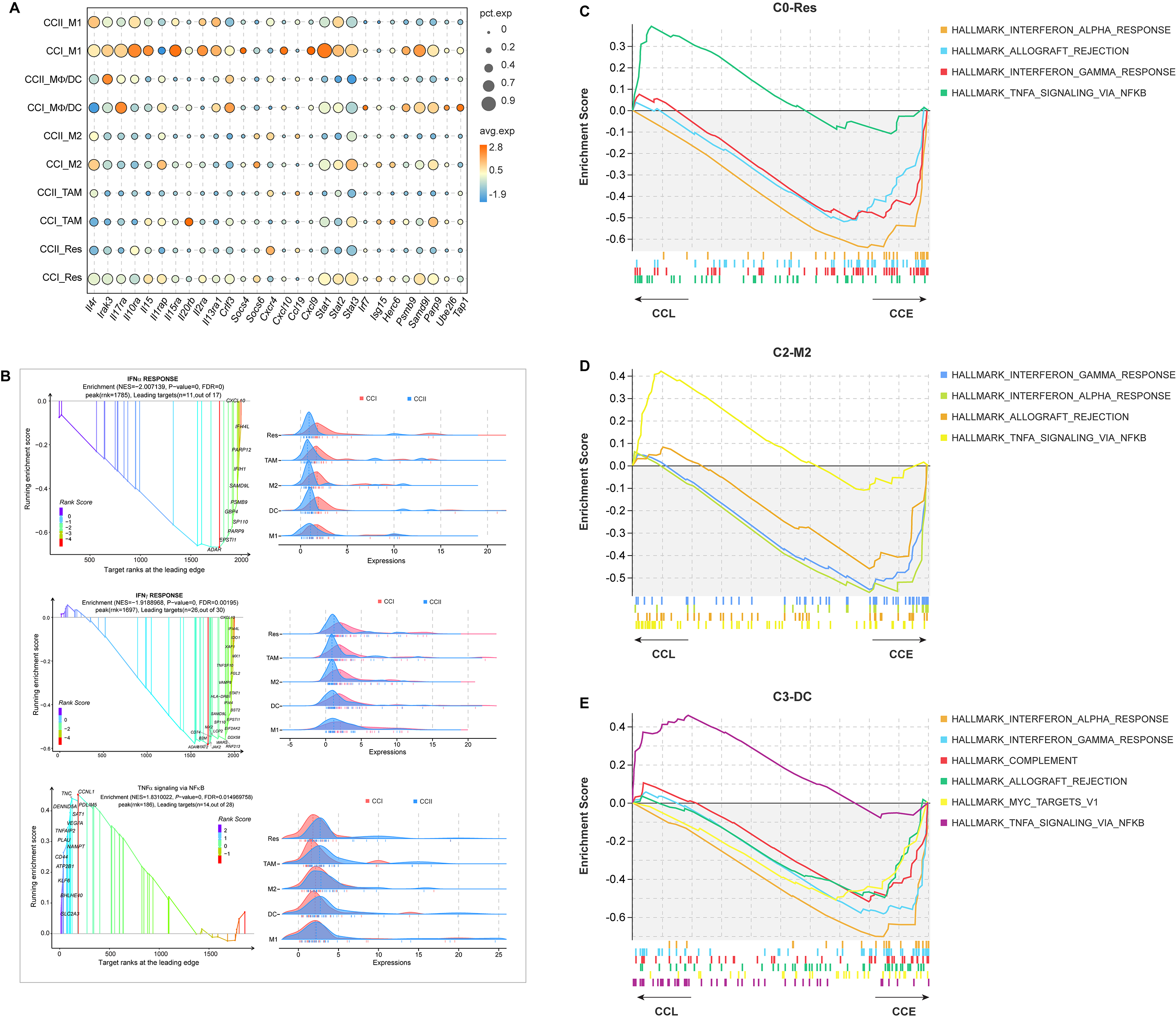

Supplement: Supplementary file 1 [file cancers-14-05785-s001.zip › Figure S4.tif]
